# In Vitro Geometry Analysis of Fenestrations in Endovascular Aneurysm Repair

**DOI:** 10.1177/15266028221079755

**Published:** 2022-02-28

**Authors:** Claire van der Riet, Richte C. L. Schuurmann, Reinoud P. H. Bokkers, Fenna A. van der Zijden, Ignace F. J. Tielliu, Cornelis H. Slump, Jean-Paul P. M. de Vries

**Affiliations:** 1 Department of Surgery, Division of Vascular Surgery, University Medical Center Groningen, University of Groningen, Groningen, The Netherlands; 2 Multimodality Medical Imaging Group, Technical Medical Centre, University of Twente, Enschede, The Netherlands; 3 Department of Radiology, Medical Imaging Center, University Medical Center Groningen, University of Groningen, Groningen, The Netherlands; 4 Faculty of Electrical Engineering, Mathematics and Computer Science (EEMCS), Robotics and Mechatronics (RAM), University of Twente, Enschede, The Netherlands

**Keywords:** in vitro model, balloon-expandable stents, geometry, target artery/vessel/branch, 3D reconstructions, computed tomographic angiography, fenestration

## Abstract

**Purpose:**

Changes in the flared end of balloon-expandable covered stent (BECS) may precede BECS-associated complications but are not regularly assessed with computed tomographic angiography (CTA) after fenestrated endovascular aneurysm repair (FEVAR). Validation of the flare geometric analysis (FGA) and assessment of intraobserver and interobserver variability are investigated in this study.

**Methods:**

Two series of 3 BeGraft BECSs (Bentley InnoMed GmbH, Hechingen, Germany) and 1 series of 3 Advanta V12 BECSs (Getinge AB, Göteborg, Sweden) were deployed in 3 side branches (45°, 60°, and 90° aortic branch angles) of an aorta phantom model. A standard post-FEVAR CTA scan was acquired. Computed tomographic angiography–derived measurements consisted of centerline reconstructions and placement of 3-dimensional coordinate markers by 2 observers in a vascular workstation. Flare geometric analysis calculates 3 BECS parameters: the circumferential flare-to-fenestration distance (FFD), which is the distance from the proximal end of the flare to fenestration, and diameters at the proximal end of the flare (Dflare) and at the fenestration (Dfenestration). Computed tomographic angiography–derived measurements were validated against microscopy measurements. Bland-Altman plots were used to determine the intraobserver and interobserver variability of the BECS parameters and intraclass correlation coefficient (ICC).

**Results:**

For each BECS, the FFD at 4 equidistant quadrants of the circumference, Dflare, and Dfenestration were calculated. The mean difference and repeatability coefficient (RC) of the validation were 0.8 (2.1) mm for FFD, 0.4 (1.0) mm for Dflare, and −0.2 (1.2) mm for Dfenestration. The mean intraobserver and interobserver difference (RC) was 0.5 (1.6) mm and 0.7 (2.6) mm for FFD, 0.1 (0.6) mm and 0.1 (0.7) mm for Dflare, and −0.1 (0.8) mm and −0.8 (1.0) mm for Dfenestration. The mean ICC of intraobserver variability was 0.86 for FFD, 0.94 for Dflare, and 0.78 for Dfenestration. The mean ICC of interobserver variability was 0.77 for FFD, 0.92 for Dflare, and 0.48 for Dfenestration.

**Conclusion:**

This study showed that FGA of the flared ends of BECS can be performed with high accuracy in a phantom model, with good intraobserver and interobserver variability. Flare geometric analysis can be used to determine flare geometry of the BECS on standard post-FEVAR CTA scans.

## Introduction

Balloon-expandable covered stents (BECS) are used in fenestrated endovascular aneurysm repair (FEVAR) to connect the fenestrated stent graft (FSG) to target arteries. This connection is secured by flaring the proximal end of the BECS with an oversized balloon. Adequate flaring is essential to secure the seal between BECS and fenestration to minimize the risk of blood leaking between the fenestration and the BECS into the aneurysm (type 3c endoleak).

Previous studies reported that 33% to 52% of all reinterventions after FEVAR are performed for complications associated with BECS, such as type 3c endoleak, BECS stenosis, BECS occlusion, or stent fracture.^1,2^ Detecting the origin of an endoleak after FEVAR can be difficult to appreciate on standard computed tomographic angiography (CTA) scans. Often additional selective angiography is necessary to detect which of the BECS is the source of a BECS-related endoleak of other complications. The ability to foresee BECS-associated complications so that they could be prevented would be beneficial. A validated method to accurately determine the geometry of the flared end of each single BECS may be helpful.^3^

Flare geometry analysis (FGA) for quantification and visualization of the flared end of the BECS on standard CTA scans was previously introduced by Overeem et al.^4^ Further improvements of the FGA and measurement protocol have been made, the method to calculate the BECS migration parameter was redesigned, and the number of coordinate markers at the proximal end of the flare and at fenestration was reduced. The aim of this study was to validate the FGA of CTA-derived measurements compared with gold standard microscopy measurements and to determine the intraobserver and interobserver variability of the upgraded FGA.

## Methods

This study was conducted with an in vitro phantom model of the aorta with 3 side branches. The study protocol consisted of the following steps:

1. Experimental setup: deployment of BECSs in the branches and flaring of the proximal ends;
2. Acquisition of a CTA scan of the model with the BECS in situ;
3. CTA-derived measurements in a vascular workstation, consisting of centerline reconstructions and placement of 3-dimensional (3D) coordinate markers;
4. FGA by dedicated software; and
5. Gold standard measurements of the flared end of the BECS by microscopy.

Two clinical examples of a BECS with and without a complication during follow-up are presented in the “Results” section.

### Experimental Setup

**Figure 1.**
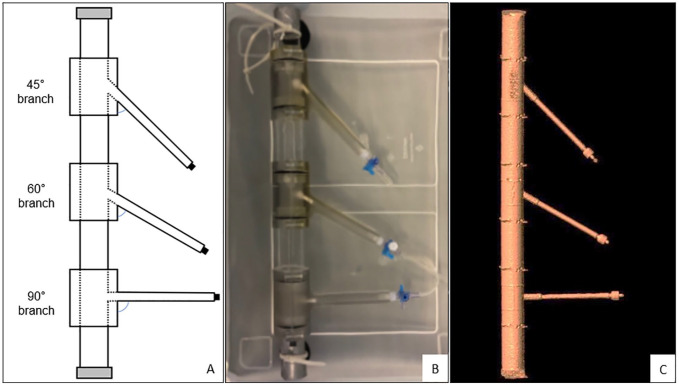
Aorta phantom model: (A) schematic view, (B) phantom model in the container filled with water before CTA scan, and (C) 3-dimensional segmentation of the contrast-rich lumen from CTA scan. CTA, computed tomographic angiography.

The rigid phantom model mimicking a straight aortic neck was designed with Solid Works SP3 software (Dassault Systèmes SolidWorks Corp., Vélizy, France). The branches were constructed of modular detachable components and manufactured of a transparent PolyJet photopolymer (VeroClear; Stratasys, Eden Prairie, Minnesota) by 3-dimensional printing with the Objet260 Connex3 printer (Stratasys) at the University of Twente (Enschede, The Netherlands) with an accuracy of ≤3 µm. The anatomical characteristics of the in vitro model, such as diameters and aortic branch angles, were based on average human anatomy.^5,6^ The phantom model had an inner aortic diameter of 26 mm and branch diameters of 6 mm. The aortic branch angles were 45°, 60°, and 90° (Figure 1).

### Deployment of BECS

A BECS was deployed in each of the 3 branches of the phantom model during each session. A total of 3 sessions were performed. During the first and second sessions, 7 × 28 mm BeGraft peripheral BECSs (Bentley InnoMed GmbH, Hechingen, Germany) were deployed in each branch. During the third session, three 7 × 22 mm Advanta V12 BECSs (Atrium Medical Corporation, Merrimack, New Hampshire) were deployed in each branch. These specific types of BECS were chosen because of their widespread use in FEVAR procedures.^7^ Each BECS was labeled after the session number and aortic branch angle (eg, the BeGraft S1.45° label described the BeGraft BECS of series 1 in the 45° branch).

All BECS were deployed under fluoroscopy in the angiography suite of the University Medical Center Groningen (Groningen, The Netherlands) by an experienced interventional radiologist (R.B.). The branches were catheterized using a 0.035-inch guidewire (Terumo Interventional Systems, Somerset, New Jersey). Each BECS was introduced from a cranial approach, deployed, and then flared with a 10- × 20-mm Armada balloon (Abbott, Lake Bluff, Illinois). The balloons were inflated up to their respective nominal pressures. The first series of BeGraft and Advanta V12 BECSs were deployed with 3 struts in the aorta lumen. The second series of BeGraft BECSs was placed with 3 struts in the aorta lumen.

### CTA Scan

Directly after implantation of each series of BECSs, the phantom model was filled with diluted Iomeron 350 contrast fluid (dilution fraction 1:20; Bracco Imaging GmbH, Konstanz, Germany), fixed to the bottom of a transparent box filled with water, and placed in the CT scanner (Figure 1). Images were acquired with a 384-slice CT scanner (Somatom Force; Siemens Healthineers, Erlangen, Germany). The CTA scan parameters were similar to conventional post-FEVAR CTA follow-up scans and included a tube voltage of 120 kV, a reference tube current of 31 mAs, and a detector collimation of 230 mm, with a pitch of 3.2 at a rotation time of 0.25 seconds. A matrix size of 512 × 512 pixels was used, with a pixel spacing of 0.34 mm. Images were reconstructed to 0.75 mm slice thickness at 0.6 mm increments using a medium-smooth convolution kernel.

### CTA-Derived Measurements

**Figure 2.**
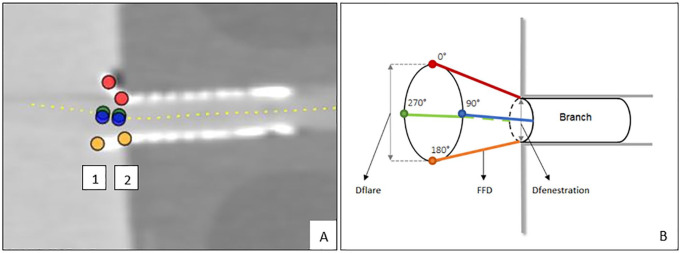
(A) Screenshot of the snake view of the centerline reconstruction through the 90° branch in the vascular workstation. Location of Cartesian coordinates at the proximal end of the flare (1) and at the fenestration (2). (B) Schematic view of location of Cartesian coordinates, Euclidean flare-to-fenestration distance (FFD), and diameter measurements at the proximal end of the flare (Dflare) and at fenestration (Dfenestration).

The measurements were performed independently by 3 observers (CR1 and RS1) using a 3mensio 10.1 Vascular workstation (Pie Medical, Bilthoven, The Netherlands). One observer performed the measurements a second time (CR2) after 1 month. A center lumen line (CLL) was constructed in the lumina of the aortas and the side branches by manual positioning of center points. Eight Cartesian coordinate markers, 4 at the proximal end of the flare and 4 at the location of the fenestration, were placed on stretched vessel reconstructions of the CLL of the branches (Figure 2). The CLLs and Cartesian coordinates were exported from the 3mensio workstation and imported into dedicated software for FGA (MATLAB, 2019b; The MathWorks Inc, Natick, Massachusetts).

### Flare Geometry Analysis

Flare geometry analysis quantified the flare geometry by means of 3 different BECS-specific parameters. The prototype FGA from a previous publication used the shadow projection method to calculate the distance from the proximal end of the flare to the shadow point on the main body. This method was replaced by the more robust Euclidean distance for 360 interpolated points over the circumference of the proximal end of the flare to its corresponding point at the fenestration (Figure 2). This method was used to introduce the flare-to-fenestration distance (FFD) as a new BECS parameter to quantify the 3D geometry.

The other 2 BECS parameters were the diameters of the proximal end of the flare (Dflare) and of the fenestration (Dfenestration). Minimum and maximum Dflare and Dfenestration were derived from the circumferences, which were determined by spline interpolation through Cartesian coordinates. Eight coordinate markers at the proximal end of the flare and at the location of the fenestration were used in the previous version of the FGA. The number of coordinate markers was reduced to 4 at each location because 8 markers in a scan with 0.75-mm slice thickness would cause too much deviation from the true circumference. The BECS parameters (FFD, Dflare, and Dfenestration) provided by FGA were compared with microscopy measurements.

### Gold Standard Microscopy Measurements

**Figure 3.**
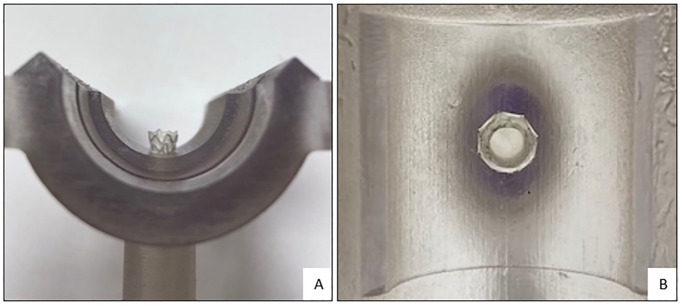
(A) Flared end of the balloon-expandable covered stent (BECS) in the 90° branch of the phantom model. (B) Look-through view of the BECS from the proximal end of the flare to fenestration.

**Figure 4.**
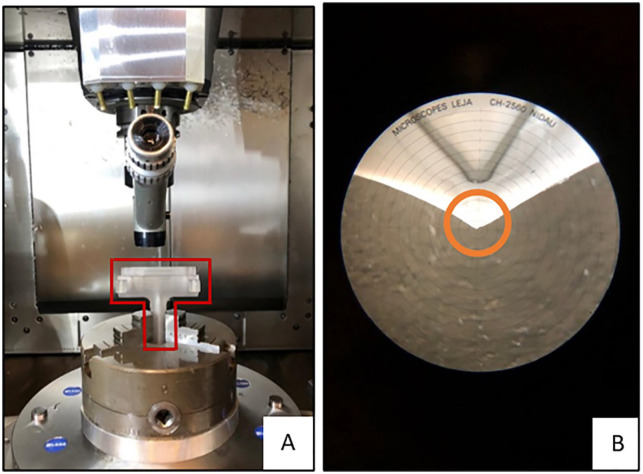
Gold standard microscopy measurements: (A) the measurement setup and the modular detachable 3-dimensional transparent component of 90° branch outlined in red, and (B) microscopy view of proximal flare and reference point 180° circled in orange.

The microcopy measurements were performed once for all BECSs per session at the Techno Centre for Education and Research (TCO) at the University of Twente (Enschede, The Netherlands). The modular detachable 3D transparent components were disassembled (Figure 3). The branches were secured in a milling machine (type C12U; Hermle Nederland BV, Horst, The Netherlands). The microscope (type NMTB40; Leja Nidau Swiss Centering, Nidau, Switzerland) was positioned in a holder above the branch and used to focus on the locations of interest at the proximal end of the flare (Figure 4). After calibration of the microscope, navigation of the phantom model to the location of interest was tracked by the milling machine with a repeatable position accuracy of ≤5 µm. Cartesian coordinates of the location were given by the milling machine.

The BeGraft BECS has 7 stainless steel struts at the proximal end of the flare, whereas the Advanta V12 BECS has 8 struts. The Cartesian coordinates of the top of the strut closest to 0°, 90°, 180°, and 270° were measured for each BECS (Figure 2). The corresponding points at the location of the fenestration could not be determined by microscopy because the flared end of the BECS obstructs the fenestration view. As an alternative, the Euclidean FFDs and Dfenestration were extracted from the Solid Works file of the phantom model. The Cartesian coordinates of the proximal end of the flare were projected onto the Solid Works file, and the Euclidean FFDs were calculated by the file. The gold standard for Dfenestration was defined as the inner diameter of the branch (6 mm) in the Solid Works file. This inner diameter was compared with the mean of the minimum and maximum Dfenestration of CTA-derived measurements. The minimum and maximum of Dflare were calculated by FGA based on the circumference of 4 Cartesian coordinates determined by microscopy.

### Data Analysis

Data were analyzed using SPSS 23 statistical software (IBM Corp, Armonk, New York). For the validation, FFD at 0°, 90°, 180°, and 270° (Figure 2), minimum and maximum of Dflare, and minimum Dfenestration were compared between microscopy measurements and CTA-derived measurements of both observers. In addition, the differences between the microscopy measurements and CTA-derived measurements for these parameters were compared between the different side branches. For the intraobserver and interobserver variability, FFD at 0°, 90°, 180°, and 270°, and the minimum and maximum of Dflare and Dfenestration were compared between both measurements of 1 observer (CR1-CR2) and between both observers (CR1-RS1), respectively. The variability was assessed by Bland-Altman plots and limits of agreement (±1.96 standard deviations around the mean difference).^8^ The intraclass correlation coefficient (ICC) for intraobserver and interobserver variability was assessed; ICC values <0.5 indicate poor reliability, values ≥0.5 and <0.75 indicate moderate reliability, and values ≥0.75 indicate good reliability.

### Clinical Examples

Examples of 2 BECS were selected from a clinical database of patients who underwent a FEVAR in the University Medical Center Groningen, the Netherlands. One BECS without post-FEVAR complications and 1 BECS with a complication were selected to show quantitative and visual differences in the FGA of the flared end of the BECSs during follow-up. The CTA-derived measurements were performed once by 1 observer (CR), and BECS parameters were calculated according to the measurement protocol of the validation experiment. The flare-to-fenestration diameter ratio was also calculated. This ratio was defined as the average Dflare divided by the average Dfenestration. A ratio of <1 describes a Dflare that is smaller than Dfenestration and a ratio of >1 describes a Dflare that is larger than Dfenestration.

## Results

Nine BECSs were deployed in the branches of the aorta phantom model during 3 separate sessions. All BECSs were 17% oversized relative to the inner diameter of the branch. The Advanta V12 S1.45° BECS had dislocated during transport between the CTA scan and microscopy measurements and was, therefore, excluded for further analysis. The FFD and diameter measurements were performed on the 6 BeGraft BECSs and remaining 2 Advanta V12 BECSs.

### Validation

**Figure 5.**
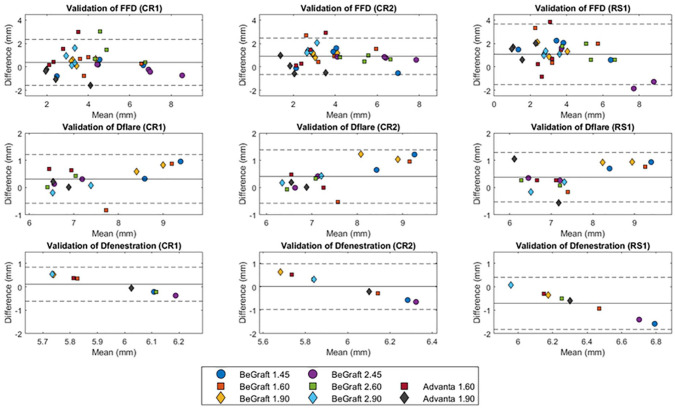
Bland-Altman plot of flare-to-fenestration distance (FFD) and the diameter at the proximal end of the flare (Dflare) and the fenestration (Dfenestration) for the gold standard microscopy measurements minus the CTA-derived measurements by CR1, CR2, and RS1. CTA, computed tomographic angiography.

Bland-Altman plots of CTA-derived measurements compared with microscopy measurements are shown per BECS parameter per observer (Figure 5). The mean differences (repeatability coefficient [RC]) for the first and second measurements of observer CR and measurements of observer RS relative to microscopy measurements were 0.4 (1.9) mm, 0.9 (1.6) mm, and 1.1 (2.6) mm for FFD, 0.3 (0.9) mm, 0.4 (1.0) mm, and 0.4 (0.9) mm for Dflare, and 0.1 (0.7) mm, 0.0 (1.0) mm, and −0.7 (1.1) mm for Dfenestration. The FFD showed a mean difference <2 mm in 84 (88%) of 96 measurements. A mean difference of <1 mm was observed in 44 (92%) of 48 Dflare measurements and in 22 (92%) of 24 Dfenestration measurements. The combined mean difference (RC) for all measurements (CR1, CR2, and RS1) compared with the microscopy measurements was 0.8 (2.1) mm for FFD, 0.4 (1.0) mm for Dflare, and −0.2 (−1.2) mm for Dfenestration.

The mean differences (RC) for the 45°, 60°, and 90° branch of the CTA-derived measurements relative to the microscopy measurements were 0.3 (0.8) mm, 1.1 (0.7) mm, and 0.8 (0.6) mm for FFD, 0.5 (0.3) mm, 0.3 (0.3) mm, and 0.4 (0.4) mm for Dflare, and 0.0 (0.3) mm, 0.2 (0.3) mm, and 0.5 (0.3) mm for Dfenestration.

### Intraobserver and Interobserver Variability

**Figure 6.**
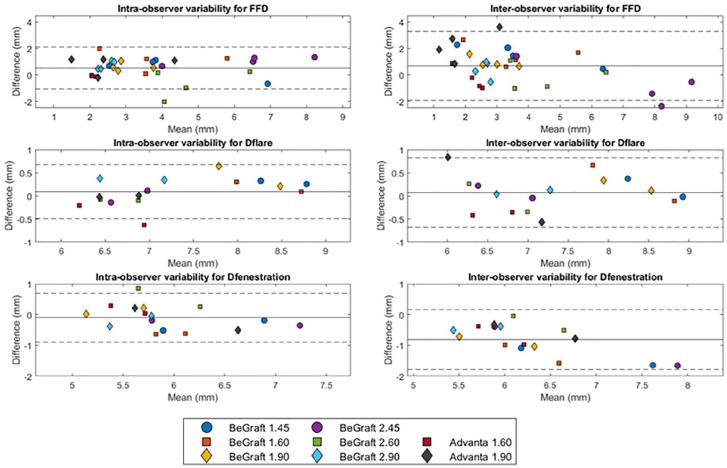
Bland-Altman plot of flare-to-fenestration distance (FFD) and the diameter at the proximal end of the flare (Dflare) and the fenestration (Dfenestration) for the intraobserver variability (CR1-CR2) and the interobserver variability (CR1-RS1).

The mean difference (RC) of intraobserver variability (CR1-CR2) was 0.5 (1.6) mm for FFD, 0.1 (0.6) mm for Dflare, and −0.1 (0.8) mm for Dfenestration (Figure 6). The mean ICC of intraobserver variability was 0.86 for FFD, 0.94 for Dflare, and 0.78 for Dfenestration. The mean difference (RC) of interobserver variability (CR1-RS1) was 0.7 (2.6) mm for FFD, 0.1 (0.7) mm for Dflare, and −0.8 (1.0) mm for Dfenestration. The mean ICC of interobserver variability was 0.77 for FFD, 0.92 for Dflare, and 0.48 for Dfenestration. The intraobserver variability was smaller than the interobserver variability for all BECS parameters. The FFD can thus be determined with a precision of ≤3.3 mm. Of 32 interobserver differences for FFD, 26 (81%) were <2.0 mm. Dflare can be determined with a precision of ≤0.8 mm and Dfenestration with a precision of ≤1.8 mm. A mean difference of <1 mm was observed in all Dflare measurements and in 11 (69%) of 16 Dfenestration measurements.

### Clinical Examples

**Figure 7.**
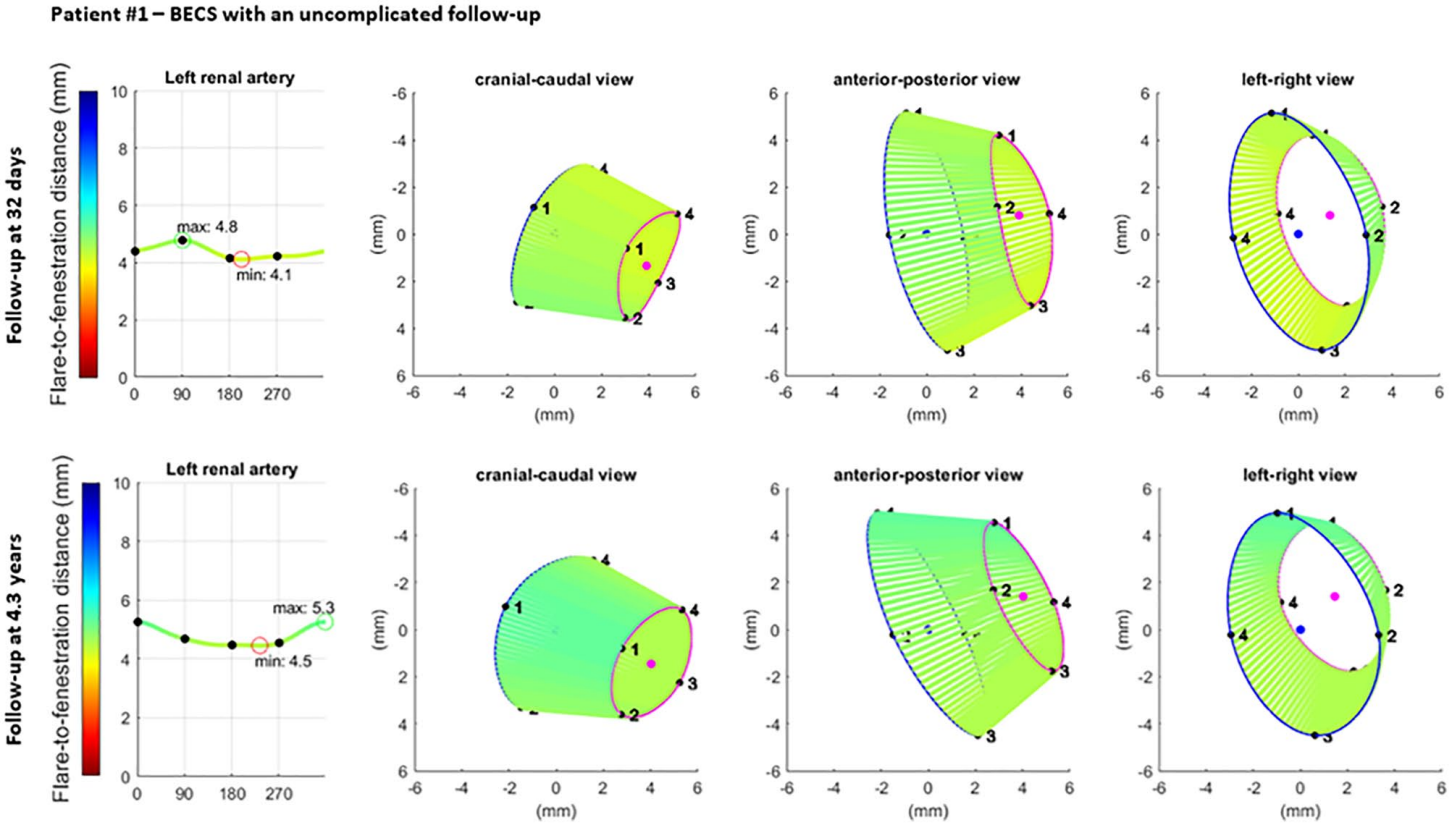
Visualization of the geometric analysis of the flared end of a BECS with an uncomplicated follow-up in the left renal artery. From left to right for 2 different follow-up moments: the flare-to-fenestration distance (FFD), and 3 views of the flare: cranial-caudal, anterior-posterior, and left-right. Blue and pink circles represent the proximal end of the flare and the location of the fenestration, respectively. The lines between the proximal end of the flare and the fenestration visualize the FFD. BECS, balloon-expandable covered stent.

**Figure 8.**
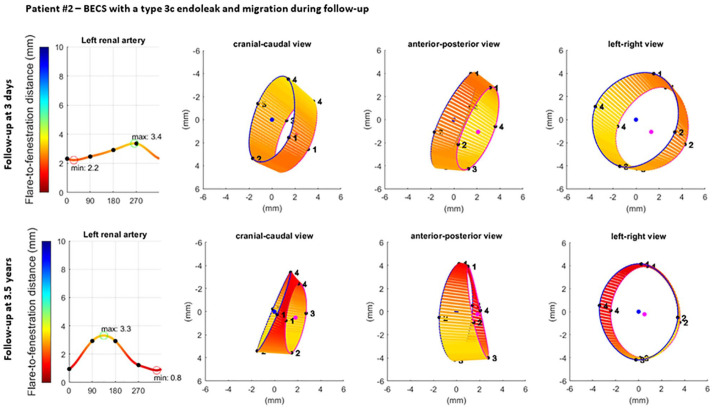
Visualization of the geometric analysis of the flared end of a BECS with a type 3c endoleak and migration in the left renal artery. From left to right for 2 different follow-up moments: the flare-to-fenestration distance (FFD), and 3 views of the flare: cranial-caudal, anterior-posterior, and left-right. Blue and pink circles represent the proximal end of the flare and the location of the fenestration, respectively. The lines between the proximal end of the flare and the fenestration visualize the FFD. BECS, balloon-expandable covered stent.

The geometric analysis of the proximal end of the flare was performed of the BECS in the left renal artery of 2 FEVAR- patients (Figures 7 and 8, respectively). The Advanta V12 BECS of patient 1 remained uncomplicated during CT follow-up of 4.3 years. At the first post-FEVAR CTA scan, the minimum and maximum FFD were 4.1 and 4.8 mm, the average Dflare and Dfenestration were 8.4 and 6.2 mm, and the flare-to-fenestration diameter ratio was 1.4. At the last available follow-up CTA scan at 4.3 years, the minimum and maximum FFD were 4.5 and 5.3 mm, the average Dflare and Dfenestration were 8.8 and 6.0 mm, and the flare-to-fenestration diameter ratio was 1.5 for the uncomplicated BECS.

The Advanta V12 BECS of patient 2 remained uncomplicated during 3.2 years of CT follow-up, but a type 3c endoleak was detected after 3.3 years. At the first post-FEVAR CTA scan, the minimum and maximum FFD were 2.2 and 3.4 mm, the average Dflare and Dfenestration were 8.4 and 7.3 mm, and the flare-to-fenestration diameter ratio was 1.2. At the follow-up CTA scan 3.3 years after the FEVAR procedure, the minimum and maximum FFD were substantially reduced to 0.8 and 3.3 mm, which led to a type 3c endoleak; the average Dflare and Dfenestration were 7.9 and 7.2 mm; and the flare-to-fenestration diameter ratio was 1.1.

## Discussion

In this study, dedicated software to determine the geometry of the flared end of BECS was validated to gold standard microscopy measurements in an in vitro model. Distances from the flared end of the BECS to the fenestration could be determined with FGA within 2 mm measurement variability, which can be considered accurate considering the CTA slice thickness of 0.75 mm. The intraobserver and interobserver variability for distance and flare and fenestration diameter measurements were good.

The aortic branch angles of the phantom model and oversizing of the BECS diameters were chosen based on suggestions in the literature.^6–9^ Visceral artery distance measurements showed good reproducibility on stretched vessel reconstructions, which were also used for the measurements in this study.^10^ The intraobserver and interobserver variability were comparable to studies reporting preoperative FEVAR and EVAR sizing measurements with use of a vascular workstation.^11,12^ In FEVAR patients, the blood flow in the fenestration depends on the geometry of the flared end of the BECS.^13,14^ The flared end of the BECS is exposed to hemodynamic forces that can cause changes in flare geometry over time.^9^

The 3D flare geometry is difficult to determine with C-arm fluoroscopy or digital subtraction angiography during the procedure. Moreover, the BECS geometry is not regularly assessed during post-FEVAR CTA follow-up. A substantial proportion of post-FEVAR reinterventions have to be performed to treat BECS-associated complications.^1,2^ Physicians often need, however, to perform selective angiography of all BECSs during the reintervention procedure to identify whether the BECS is causing the problem. Flare geometry analysis may be helpful to detect BECS-associated complications to plan a reintervention and limiting radiation exposure and contrast use.

The clinical examples in this study showed the added value of FGA for the occurrence of a type 3c endoleak. The complicated BECS had a smaller flare-to-fenestration ratio than the uncomplicated BECS on the first postoperative CTA scan, and this ratio continued to decrease during follow-up. This corresponds with previous findings where BECSs with a type 3c endoleak had a flare-to-fenestration ratio of <1.1 or decreasing flare-to-fenestration ratio during follow-up.^4^ Furthermore, the minimum FFD was shorter for the complicated BECS and decreased further during follow-up. Flare geometry analysis visualized that the BECS slowly migrated into the target artery, which is difficult to determine during standard CTA assessment. One of the advantages of the FGA is that the measurements can be extracted from standard follow-up CTA scans, and no extra series have to be made for this purpose.

One limitation of this study is the absence of an FSG in the phantom model. The radiopaque markers around the fenestration of the FSG may affect the accuracy of the analysis. A second limitation is that the phantom model is a simplified representation of a human aorta. The centerline reconstruction and CTA-derived measurements in vivo may be less accurate than in vitro due to anatomical irregularities. Third, the Dfenestration for the gold standard was derived from the inner diameter of the branch. Therefore, the gold standard of Dfenestration may be overestimated, which may explain why Dfenestration was slightly underestimated compared with Dflare. Fourth, the validation was only performed with BeGraft and Advanta V12 BECSs. Further research is needed to verify whether this method is equally accurate for other types of BECS. A study with a large series of FEVAR patients with and without complications should be conducted to determine whether this accuracy is accurate enough to detect clinically relevant changes in BECS geometry, the relationship between changes in BECS geometry and BECS-associated complications, and the true clinical merit of this FGA.

## Conclusion

This study showed that FGA of the flared ends of BECS can be performed with high accuracy in a phantom model, with good intraobserver and interobserver variability. Flare geometry analysis can be used to determine flare geometry of the BECS on standard post-FEVAR CTA scans. However, a large BECS series derived from post-FEVAR CTA scans with and without complications is needed to determine its real clinical added value.
